# Elevated plasma levels of IP-10 and MIG are early predictors of loss of control among elite HIV controllers

**DOI:** 10.3389/fimmu.2024.1446730

**Published:** 2024-08-29

**Authors:** Eva Poveda, Wendy Fitzgerald, Jacobo Alonso-Domínguez, José Aguayo-Arjona, Ana Mariño, Hortensia Álvarez, Nieves Valcarce, Alexandre Pérez, Ezequiel Ruiz-Mateos, Leonid Margolis, Michael M. Lederman, Michael L. Freeman

**Affiliations:** ^1^ Group of Virology and Pathogenesis, Galicia Sur Health Research Institute (IIS Galicia Sur), SERGAS-UVIGO, Vigo, Spain; ^2^ Section on Intercellular Interactions, Eunice Kennedy Shriver National Institutes of Child Health and Human Development, National Institutes of Health, Bethesda, MD, United States; ^3^ Statistics and Methodology Unit, Galicia Sur Health Research Institute (IIS Galicia Sur), SERGAS-UVIGO, Vigo, Spain; ^4^ Complexo Hospitalario Universitario de Ferrol, Sergas, A Coruña, Spain; ^5^ Institute of Biomedicine of Seville (IBiS), Virgen del Rocío University Hospital, CSIC, Clinical Unit of Infectious Diseases, Microbiology and Parasitology, University of Seville, Seville, Spain; ^6^ Faculty of Natural Sciences and Medicine, Ilia State University, Tbilisi, Georgia; ^7^ Department of Medicine, Case Western Reserve University, Cleveland, OH, United States

**Keywords:** HIV, elite controllers, IP-10, MIG, functional cure

## Abstract

Plasma cytokine levels were quantified among 30 persons with HIV (PWH) identified as elite controllers (15 transient controllers [studied a median of 1.38 years before losing viral control] and 15 persistent controllers). Thirty antiretroviral therapy (ART)-naive PWH, 30 ART-treated PWH with undetectable viremia, and 30 HIV-uninfected controls also were studied. Higher levels of cytokines were recognized among PWH than among controls, with EC displaying the highest levels. Elevated levels of IP-10 and MIG were identified among transient controllers as predictors of the loss of viral control. These findings offer feasible biomarkers for predicting virologic outcome and loss of control in EC.

## 1 Introduction

HIV infection continues to pose a significant global health challenge, with over 39 million persons with HIV (PWH) worldwide (1). Antiretroviral therapy (ART) has been a major breakthrough in managing the virus and improving the quality of life for PWH. However, achieving a complete cure remains elusive, and long-term treatment is required to suppress viral replication and prevent disease progression in most individuals. Among PWH, elite controllers (EC) are an exceptional population (<1%) of individuals who have a remarkable ability to naturally maintain undetectable or very low viral loads (<50 copies/ml) for extended periods without the need of ART (2–4), offering unique insights into potential functional cure strategies (5, 6).

EC are a heterogeneous population defined differently based on the follow-up length, viremia detection limits, presence/absence of blips, and/or CD4 T cell counts. At least two well-defined phenotypes have been identified (7, 8). The first group, known as persistent controllers (PC), maintain HIV plasma levels below the limits of detection by standard assays in the absence of blips on a continuous and durable basis. The second group, known as transient controllers (TC), eventually lose control of virus replication.

In this study, we investigated the plasma cytokine profile in well-characterized cohorts of PWH, including TC and PC, ART-naive and ART-treated PWH, and HIV-uninfected controls. We identified specific cytokines in plasma that could serve as biomarkers for distinguishing these groups and potentially predicting virologic control outcomes, and more interestingly, the loss of control among TCs. This research adds new relevant knowledge to the understanding of the immunological mechanisms underlying HIV control and the pursuit of functional cure strategies.

## 2 Material and methods

### 2.1 Sample selection and preparation

Frozen plasma samples were obtained from 120 enlisted participants. One group included 30 individuals newly diagnosed with HIV and not yet on ART, aged 41 [32-52] years, with a median [IQR] viral load (VL) of 27450 [9700-110000] HIV-RNA copies/ml and 456 [267-657] CD4 T cells per microliter. Another 30, with an age of 52 [43-56] years, had been on ART for a median of 9 years [IQR], maintaining nondetectable viremia and a median of 827 [609-1071] CD4 T cells per microliter, allowing us to assess the effects of long-term treatment on the plasma cytokine profile. Another group of participants were 30 elite controllers (EC), who naturally suppressed HIV replication (<50 copies/ml) for a median of 14.4 years [IQR]. Their main characteristics included an age of 53 [43-58] years, and 830 [520-1077] CD4 per microliter. Among the EC, 15 were persistent controllers (PC), consistently maintaining HIV control and no CD4 T-cell decline and 15 were transient controllers (TC), studied a median of 1.38 years [IQR: 0.95-2.88] before the loss of control. Loss of control was defined as two consecutive VL measurements above the detection limit (>50 HIV-RNA copies/ml) one year apart and/or CD4 T-cell decline during the follow-up period (a statistically significant and negative CD4 count slope, evaluated by linear regression, non-zero slope, p < 0.05), as previously reported (7). Eight of the 15 TC individuals correspond to those who have lost control of virus replication, while the other seven are individuals who experienced a decline in CD4 T cell counts. At the time of the loss of control, median VL was 140 [54-557] copies/ml and CD4 T cell counts decreased from 834 [640-1038] to 518 [367-668]. Finally, 30 individuals without HIV served as controls (36 [29-41] years old and 842 [712-1018] CD4 per microliter), offering a benchmark for cytokine profiles in the absence of HIV infection (more detailed demographic information can be found in **Supplementary Table 1**). The study was approved by the regional Ethics committee and external scientific and ethics committee of the HIV Biobank (Spanish HIV/AIDS Research Network).

### 2.2 Cytokine quantification

We quantified levels of 39 cytokines and chemokines in plasma using a multiplexed bead-based Luminex assay, namely interleukin (IL)-1α, IL-1β, IL-3, IL-4, IL-6, IL-7,IL-8, IL-9, IL-10, IL-12p70, IL-13, IL-15, IL-16, IL-17, IL-18, IL-21, IL-22, IL-33, interferon (IFN)-α, IFN-β, IFN-γ, IFN-λ, monocyte chemoattractant protein-1 (MCP-1 or CCL2), macrophage inflammatory protein-1α (MIP-1α or CCL3), MIP-1β (CCL4), MIP-3α (CCL20), RANTES (CCL5), eotaxin (CCL11), growth-regulated alpha (GRO-α or CXCL1), monokine induced by IFN-γ (MIG or CXCL9), IFN-γ-induced protein 10 (IP-10 or CXCL10), IFN-inducible T-cell alpha chemoattractant (ITAC or CXCL11), tumor necrosis factor-α (TNF-α), TNF-related apoptosis-inducing ligand (TRAIL), granulocyte-macrophage colony-stimulating factor (GM-CSF), macrophage colony-stimulating factor (M-CSF), transforming growth factor-β (TGF-β) and calgranulin A (Calg A). These immune signaling proteins were selected based on their potential relevance to HIV pathogenesis and immune response.

Antibody pairs and protein standards were procured from R&D Systems, with the exception of those utilized for IL-3, IL-4, IL-9, IFN-α (sourced from Biolegend, San Diego, CA), and IL-21 (obtained from Thermo Fisher, Waltham, MA). Rigorous verification ensured the absence of cross-reactivity. MagPlex microspheres from Luminex (Austin, TX, USA), featuring distinctive spectral signatures, were coupled with cytokine-specific capture antibodies following the manufacturer’s guidelines.

Plasma samples were thawed on ice for use in multiplexed bead-based assays. Subsequently, plates were subjected to analysis on a Luminex 200 instrument, with data interpretation facilitated by the Bio-plex Manager Software (Bio-Rad, Hercules, CA, USA). Cytokine concentrations were determined using 5P regression algorithms.

### 2.3 Statistical analysis

We conducted statistical analyses using SPSS (v26.0) and RStudio (v2023.09.0 + 463). A significance level of p < 0.05 was considered significant.

Continuous variables were presented as medians and interquartile ranges (IQR), while categorical variables were expressed as percentages. The chi-square test was used for qualitative variables to compare between groups. Normality was assessed using the Shapiro-Wilk test for continuous variables, since the sample size was less than 50 individuals per group. The bivariate analyses used were the Mann-Whitney test when the comparison was between two groups (as in the case of controls vs. PWH) and the Kruskal-Wallis test when the comparison was among at least three groups, followed by Dunn’s multiple comparisons test for continuous variables.

Receiver operating characteristic (ROC) analysis was carried out to assess the specific contribution of each cytokine. Youden’s index (J) was employed to define cut-off values, with the formula *J = sensitivity + specificity - 1* guiding the selection process. Youden’s index was computed for all points on the ROC curve, with the maximum value serving as the criterion for determining the optimal cut-off for each cytokine.

Multivariate analysis, including random forest analysis and principal component analysis (PCA), was employed to discern patterns and relationships within the complex dataset. Random forest analysis was utilized to identify key cytokines contributing to the classification of patient groups, providing insights into the most influential factors. PCA, on the other hand, served to reduce the dimensionality of the data, allowing for a more comprehensive exploration of underlying patterns and correlations among cytokine variables.

## 3 Results

### 3.1 Levels of cytokines in plasma distinguish PWH groups

**Figure 1:**
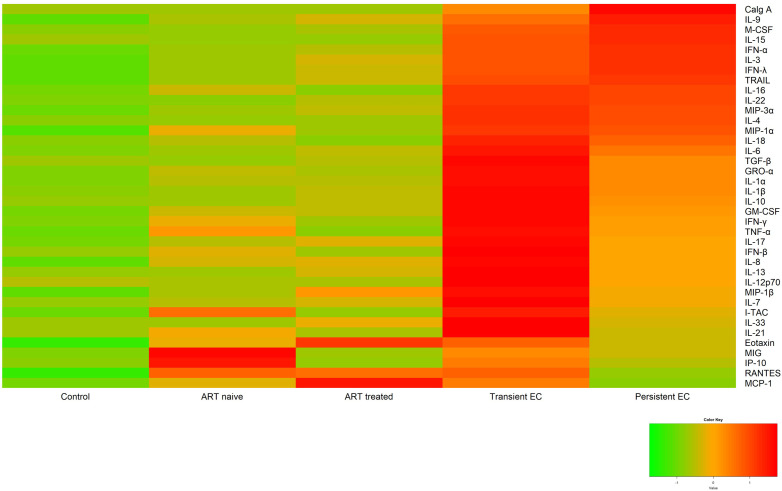
Heatmap of the relative plasma cytokine levels. The levels of cytokines for each study group are represented as the standardized mean of each group as a comparator for each cytokine.

We conducted an analysis of 39 cytokines in plasma samples obtained from the previously described populations. The cytokines that best differentiate the groups are listed in **Table 1**. Overall, the relative median levels of cytokines in plasma were 2.72-fold higher for PWH than for the control group. Within the PWH group, EC exhibited higher plasma cytokine levels than those on ART and those who have not yet started treatment (naive), 2.31 and 2.48-fold, respectively. Within the EC group, TC had cytokine values 1.68-fold higher than PC. **Figure 1** depicts the heatmap illustrating the concentrations of the plasma cytokines for each group by their z-scores (**Supplementary Figure 1** contains this same heatmap with the z-score values in each of the cells).

**Table 1:** Specific plasma cytokine levels as biomarkers of PWH groups.

Cytokines	Groups (median pg/ml)			*p*-value^A^	AUC	Sen/Spe	Cut-off (pg/ml)
	PWH	vs	HIV-				
**MIG**	1673.69 [IQR: 1128.64-3723.24]		640.64 [IQR: 504.39-989.93]	<0.001	0.841	76.7/83.3	1114.975
**ITAC**	41.85 [IQR: 6.37-134.40]		6.37 [IQR: 6.37-6.37]	<0.001	0.784	58.9/93.3	16.66
**IL-8**	4.09 [IQR: 2.40-6.97]		1.53 [IQR: 0.50-3.59]	<0.001	0.783	85.6/63.3	1.905
**IP-10**	3031.17 [IQR: 1593.65-5402.02]		1425.55 [IQR: 868.58-1848.02]	<0.001	0.769	66.7/83.3	1915.12
**RANTES**	28889.86 [IQR: 15206.84-58281.60]		7744.10 [IQR: 4968.81-23179.21]	<0.001	0.756	83.3/63.3	10841.035
**MCP-1**	11.87 [IQR: 5.61-20.40]		3.55 [IQR: 1.05-7.88]	<0.001	0.761	55.6/90.0	9.81
**IL-18**	17.57 [IQR:10.94-36.54]		9.97 [IQR: 7.15-15.69]	<0.001	0.742	68.9/70.0	12.86
	EC	vs	ART-naïve				
**IL-22**	13.86 [IQR: 10.26-19.52]		2.00 [IQR: 2.00-2.00]	<0.001	0.828	83.3/80.0	7.275
**TGF-β**	46.21 [IQR: 14.75-73.67]		6.41 [IQR: 0.84-21.43]	<0.001	0.823	86.7/63.3	8.62
**IL-15**	9.96 [IQR: 6.49-14.36]		3.16 [IQR: 1.70-5.57]	<0.001	0.807	76.7/80.0	6.315
	EC	vs	ART-treated				
**IL-18**	45.87 [IQR: 23.78-105.00]		11.76 [IQR: 8.16-15.86]	0.036	0.911	80.0/100.0	19.79
**TNF-α**	5.66 [IQR: 3.51-8.77]		1.25 [IQR: 0.56-3.74]	0.003	0.831	96.7/66.7	1.79
	TC	vs	PC				
**IP-10**	4744.80 [IQR: 2604.83-7651.70]		2068.26 [IQR: 1446.57-3770.46]	0.275	0.760	93.3/53.3	2082.185
**MIG**	2641.86 [IQR: 1305.06-4471.00]		1470.03 [IQR: 1164.44-2007.11]	1.000	0.711	60.0/86.7	2212.825

^A^The p-value was obtained using the Mann-Whitney test.

The standardized means and distributions of each cytokine were compared across the different groups. This analysis revealed statistically significant differences in cytokine levels between nearly all groups. The sole exception was the comparison between ART-naive and ART-treated groups, where no significant difference was observed, likely due to early sampling (median of 7 days) in the group without antiretroviral treatment.

### 3.2 Distinct pro-inflammatory cytokine profile in elite controllers: IP-10 and MIG are predictors of loss of control in EC

Overall, elevated levels of MIG in plasma proved to be the most reliable differentiator between PWH and uninfected controls, with median levels of 1674 vs. 641 pg/ml and an area under the curve (AUC) of 0.841, according to ROC analysis. After using the Youden’s index to choose the best cut-off point for the classification between the two groups, the value of 1115 pg/ml had a sensitivity of 76.7% and a specificity of 83.3% in classifying individuals into these groups (**Supplementary Figure 2A**).

When comparing untreated individuals (i.e., naive and EC), the most distinguishing cytokine was IL-22 with an AUC of 0.828. The cut-off using Youden’s index, 7.3 pg/ml yielded a sensitivity of 83.3% and a specificity of 80% in classifying these two phenotypes. To check a possible bias due to difference in the duration of the infection between the two groups, a correlation was made between the cytokine levels and the duration of infection, and no statistical significance was found.

In the case of individuals with controlled viremia, (ART-treated and EC), elevated levels of IL-18 distinguished them with an AUC of 0.911. More precisely, the cut-off value calculated to differentiate the two groups was 19.8 pg/ml that correctly classified 100% of the participants who exceeded this value in the EC group. Specificity was 80% (**Supplementary Figure 2B**).

MIG and IP-10 could discriminate between TC and PC phenotypes. Although the comparison of the medians of both molecules did not show significant differences, their distributions by histograms represent a much narrower variability among PC than in TC (**Supplementary Figure 3**). With AUCs of 0.711 and 0.760, respectively, cutoffs were calculated for both cytokines —2082 pg/ml for IP-10 and 2212 pg/ml for MIG. These had a sensitivity of 60.0 and 93.3% and a specificity of 86.7 and 53.3%, respectively (**Supplementary Figures 2C, D**).

Within the EC group, 93.3% of individuals with IP-10 levels equal to or greater than 2082 pg/ml (n = 14) were accurately classified as TC. Indeed, both cytokines have shown a positive correlation irrespectively of the study groups: for PWH (correlation coefficient: 0.673, p < 0.01), for ART-naive and ART-treated (CC: 0.686, p < 0.01) or for PC and TC (CC: 0.607, p < 0.01). Figures depicting the random forest, PCA and ROC curves for cytokines that best differentiated the groups are shown in **Supplementary Figures 4**-**7**.

## 4 Discussion

Our study highlights the distinct pro-inflammatory cytokine profile observed in EC, a rare subgroup of PWH capable of naturally controlling HIV replication. EC exhibited significantly higher cytokine levels than did other PWH groups, with even higher levels observed in those who later lost virologic control (TC). Within the EC group, TC displayed higher levels of MIG and IP-10 (studied 1.38 years before loss of virologic control) than did PC offering these plasma cytokines as potential biomarkers for predicting the loss of virologic control in the absence of treatment. The validation of these results opens the door for monitoring plasma levels of these predictors that might guide more frequent virologic monitoring or even consideration of study to determine if earlier administration of antiretroviral drugs to TC expected to lose virologic control confers benefit to reservoir measurements or other indices such as the proteins reported in this study. These findings are consistent with our recent demonstration in a longitudinal study among a cohort of PWH initiating ART that IP-10 and MIG levels were directly correlated with plasma HIV load (9).

Furthermore, the higher levels of IP-10 seen in TC than among PC may have implications similar to those observed in a longitudinal study evaluating the impact of ART on IP-10. In that study, the lack of reduction in IP-10 levels during ART was linked to immunological treatment failure (10). In both scenarios, this increase in IP-10 could predispose the cellular environment in a way that culminates in a virological loss such as that suffered among TC in our study. This is in agreement with a study of HIV-1 exposed seronegative individuals that suggested that reduced levels of IP-10 and MIG may favor an immune environment that is not conducive to HIV infection. This would be in line with the increased of IP-10 and MIG levels observed in our study among TC prior to their loss of virological control (11).

IL-18 may also play an important role during HIV infection. In an earlier study, we observed how higher levels of extracellular vesicle (EV)-associated IL-18 distinguished EC from ART-treated persons with suppressed viremia displaying an AUC of 0.942. In that study, after establishing a cutoff of 2.2 pg/ml, of the 22 participants with EV-associated IL-18 levels equal to or above that cutoff point, this marker correctly classified all of them as EC (12). In the present study, higher levels of IL-18 measured directly in plasma could also distinguish EC from ART-treated participants, with a cutoff value of 19.8 pg/ml, also correctly classifying 100% of EC. These results differ somewhat from the results obtained by Côrtes et al. (13), where plasma levels of IL-18 in PC tended to be lower than those in ART-treated individuals and in TC. They found that plasma IL-18 levels were similar in EC and uninfected individuals as we did. The differences observed in both studies might be explained in part for the different criteria for PC and TC classification and other characteristics within the study cohorts.

There is limited information regarding the role of IL-22 in HIV pathogenesis. In our study, in absence of ART, IL-22 shows the highest values in EC and can discriminate this group from ART-naive. This cytokine may have a protective role in HIV infection: in a study of serodiscordant couples, HIV-negative persons with higher levels of IL-22 maintained their uninfected status, despite being repeatedly exposed to HIV (14). One cell type that produces IL-22 is T helper 17 (Th17) cells, which are induced by the presence of TGF-β (another cytokine that best differentiates between EC and ART-naive in our study) and IL-6.

We would like to address some limitations of this study. While these are very well-characterized cohorts, the sample size of each group is small which might limit the power of the study or its broader applicability. The identified cutoff values might not be clinically applicable since the concentrations of the cytokines measured by the Luminex technology may differ from those obtained using other methods. Thus, further research is needed to validate these cytokine biomarkers in other cohorts and to explore their potential clinical utility in predicting HIV control and optimizing patient care.

In addition, it is important to note that cytokine assessment could not be performed in the ART-naive group later in the disease because of the test-and-treat principle, whereby the median number of days from diagnosis to ART initiation was 17 [6-67]. Interestingly, for ART-treated, who were diagnosed about 14 years earlier of the sample collection, they had a median number of days between diagnosis and ART initiation of 129.5 [17.75-780.25], indicating a considerable improvement in the achievement of the aforementioned principle. Accordingly to previous studies (15, 16), our results also support a significant decrease regarding some specific cytokines (i.e. IL-18, IP-10 and MIG) between the ART-naïve and the ART-treated stage.

In conclusion, we identified elevated plasma levels of IP-10 and MIG as early predictors of loss of control among EC. Further study of these cytokines may offer opportunities for intervention as well as insight into pathogenesis.

## Data Availability

The raw data supporting the conclusions of this article will be made available by the authors, without undue reservation.

## Supplementary material

Supplementary Figure 1 Heatmap of the relative plasma cytokine levels and their standardized means. The levels of cytokines for each study group are represented as the standardized mean of each group as a comparator for each cytokine.

Supplementary Figure 2 Decision Tree Analysis. **(A)** Comparing PWH and controls using MIG as a biomarker, **(B)** Among those with undetectable viremia using IL-18 as a biomarker, **(C)** and **(D)** within the elite controllers, IP-10 and MIG, respectively, as a differential biomarker between TC and PC groups.

Supplementary Figure 3 Distribution of IP-10 and MIG levels in elite controllers according to their status. **(A)** IP-10 levels in PC and TC. **(B)** MIG levels in PC and TC.

Supplementary Figure 4 Identification of cytokines allowing discrimination between PWH and controls. **(A)** Random forest analysis. (**B)** PCA Score plot. **(C)** Receiver operator Curve-ROC. (**D)** Best discriminating cytokines signatures.

Supplementary Figure 5 Identification of cytokines allowing discrimination between elite controllers and naive. **(A)** Random forest analysis importance plot. **(B)** PCA Score plot. **(C)** Receiver operator Curve-ROC. **(D)** Best discriminating cytokines signatures.

Supplementary Figure 6 Identification of cytokines allowing discrimination between elite controllers and ART-treated. **(A)** Random forest analysis importance plot. **(B)** PCA Score plot. **(C)** Receiver operator Curve-ROC. **(D)** Best discriminating cytokines signatures.

Supplementary Figure 7 Identification of cytokines allowing discrimination between transient and persistent controllers. **(A)** Random forest analysis importance plot. **(B)** PCA Score plot. **(C)** Receiver operator Curve-ROC. **(D)** Best discriminating cytokines signatures.
