# Supplementary figures and images for: Elevated plasma levels of IP-10 and MIG are early predictors of loss of control among elite HIV controllers
Source: Front Immunol. 2024 Aug 29;15:1446730. doi: 10.3389/fimmu.2024.1446730 (PMC11390527; doi:10.3389/fimmu.2024.1446730)

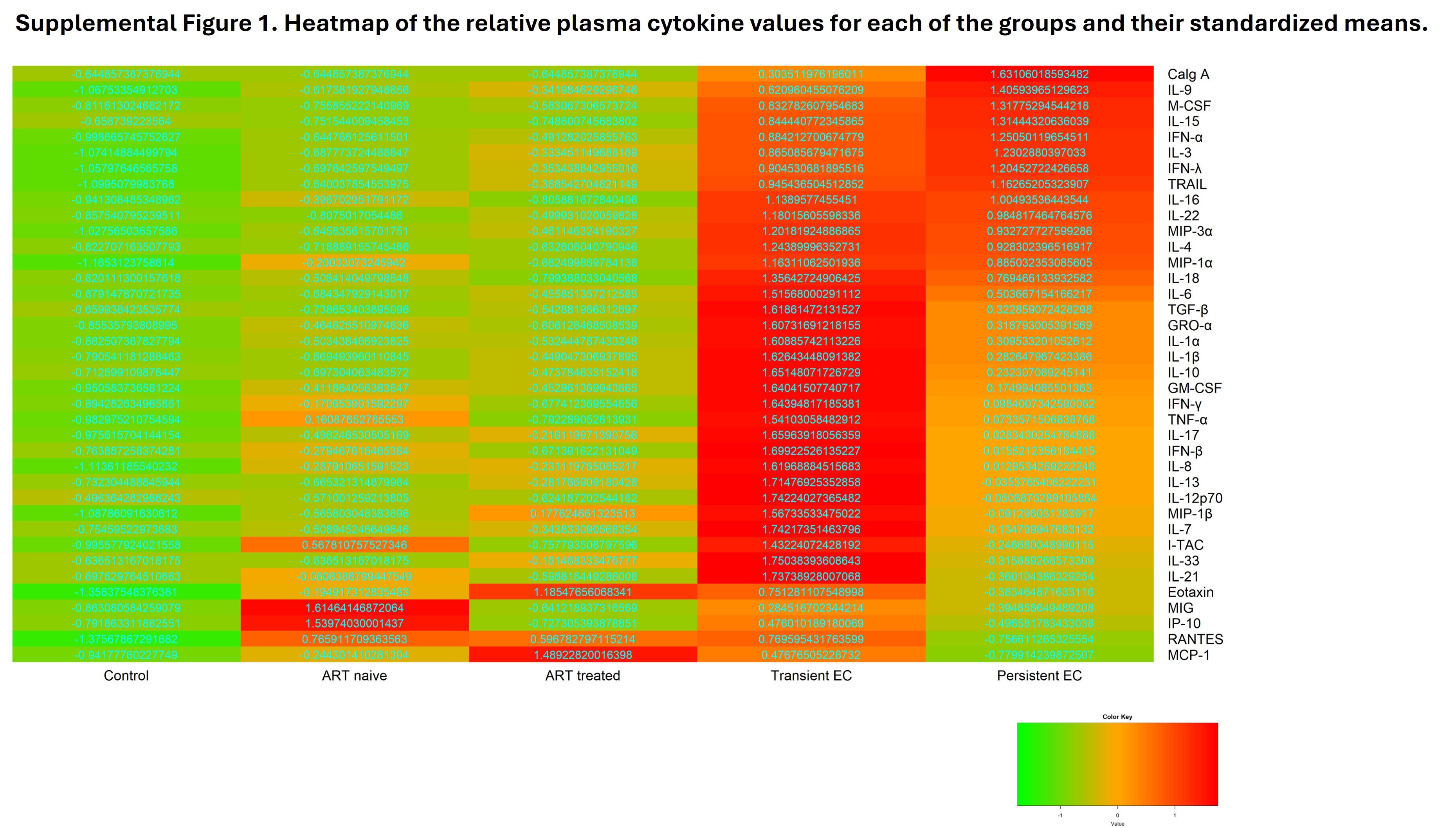

Supplement: Supplementary Figure 1 — Heatmap of the relative plasma cytokine levels and their standardized means. The levels of cytokines for each study group are represented as the standardized mean of each group as a comparator for each cytokine. [file Image1.jpeg]

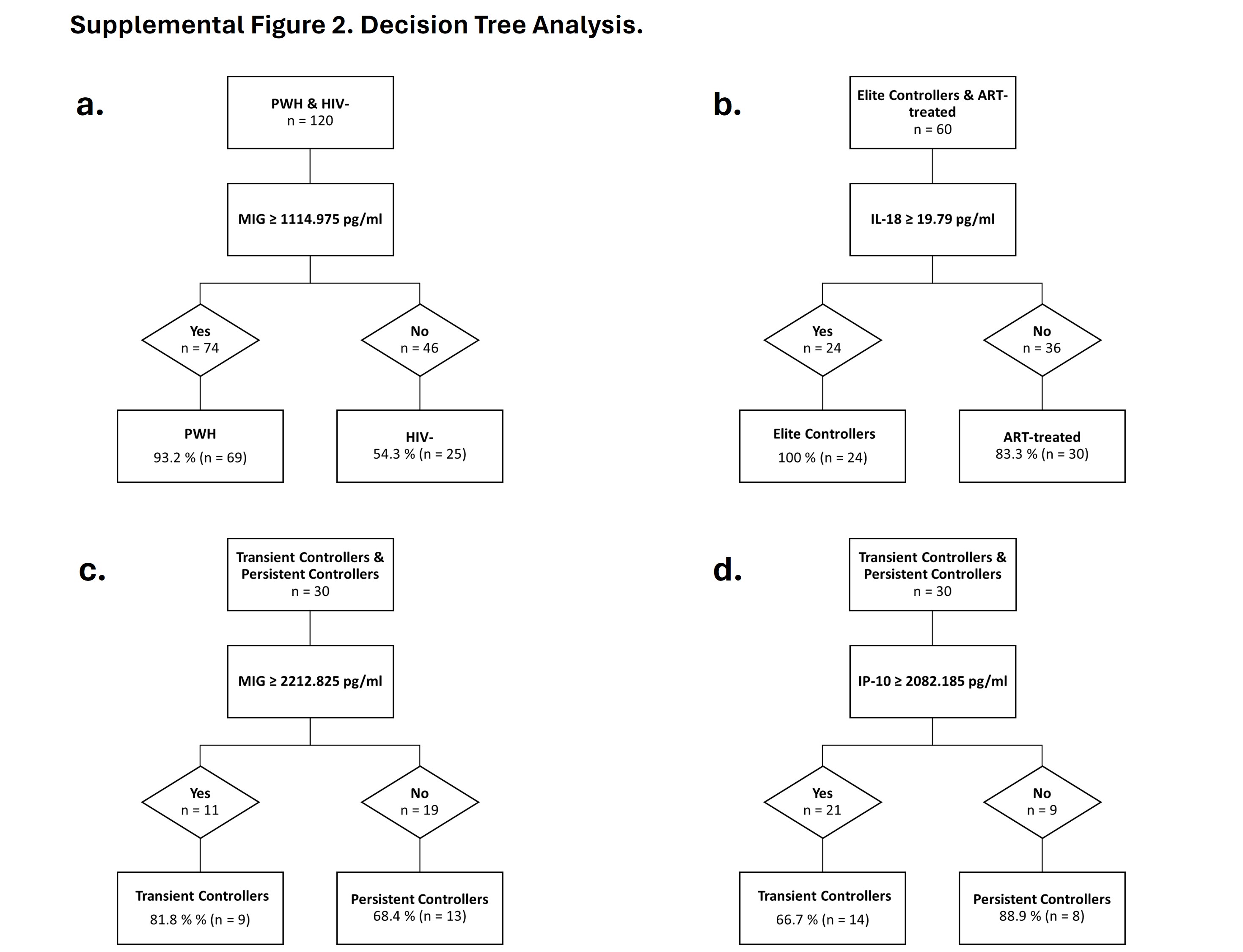

Supplement: Supplementary Figure 2 — Decision Tree Analysis. (A) Comparing PWH and controls using MIG as a biomarker, (B) Among those with undetectable viremia using IL-18 as a biomarker, (C) and (D) within the elite controllers, IP-10 and MIG, respectively, as a differential biomarker between TC and PC groups. [file Image2.jpeg]

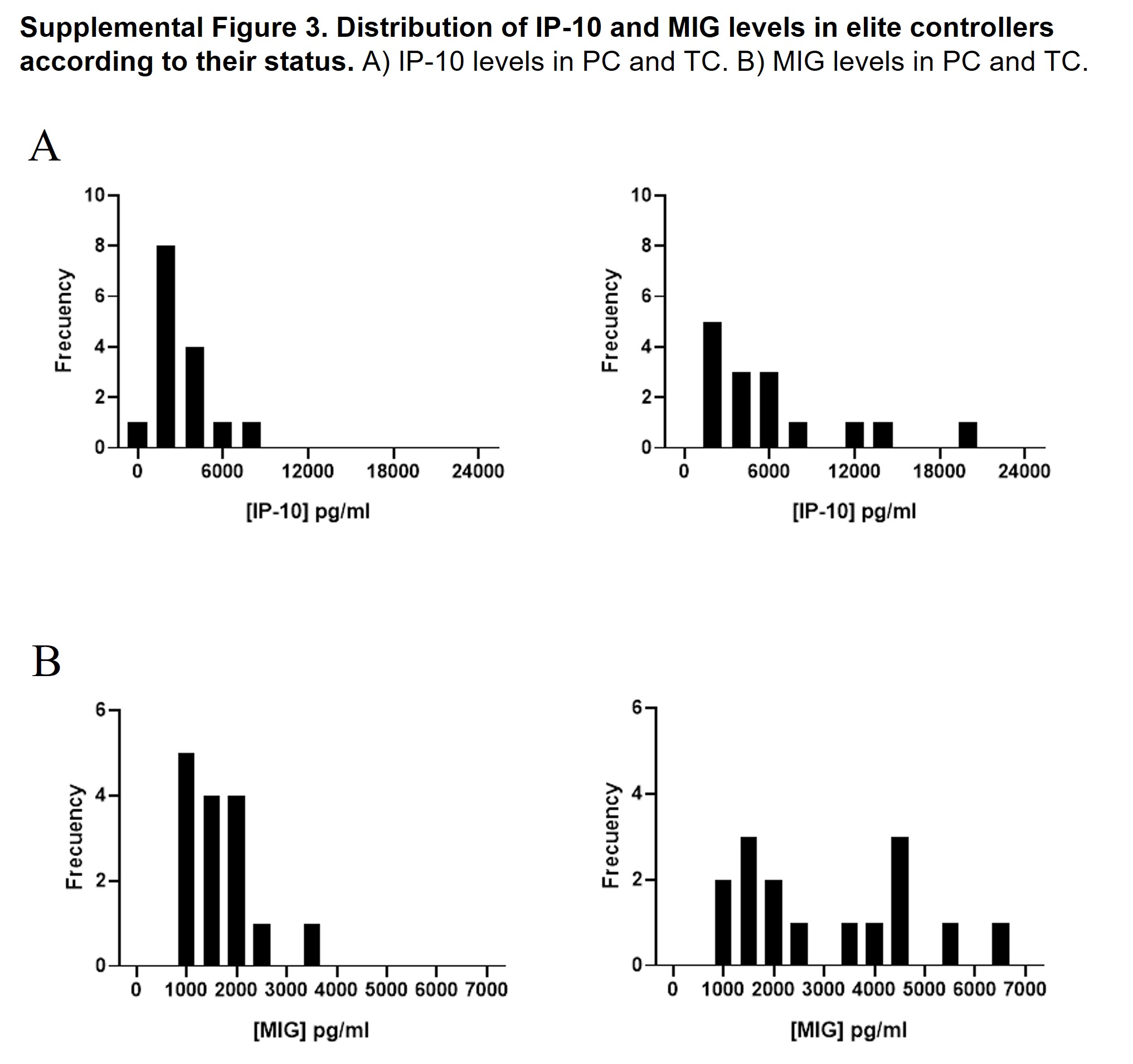

Supplement: Supplementary Figure 3 — Distribution of IP-10 and MIG levels in elite controllers according to their status. (A) IP-10 levels in PC and TC. (B) MIG levels in PC and TC. [file Image3.jpeg]

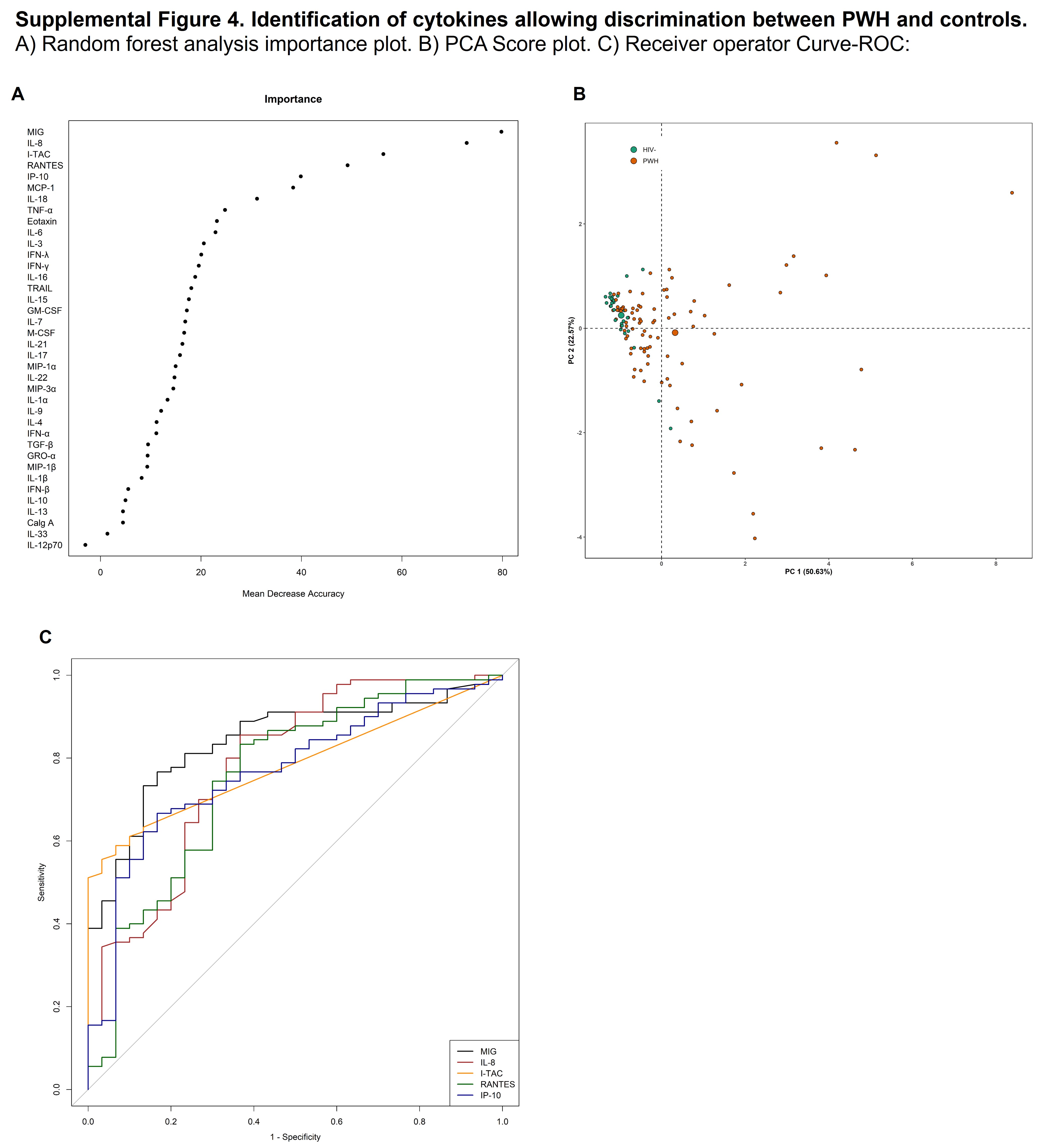

Supplement: Supplementary Figure 4 — Identification of cytokines allowing discrimination between PWH and controls. (A) Random forest analysis. (B) PCA Score plot. (C) Receiver operator Curve-ROC. (D) Best discriminating cytokines signatures. [file Image4.jpeg]

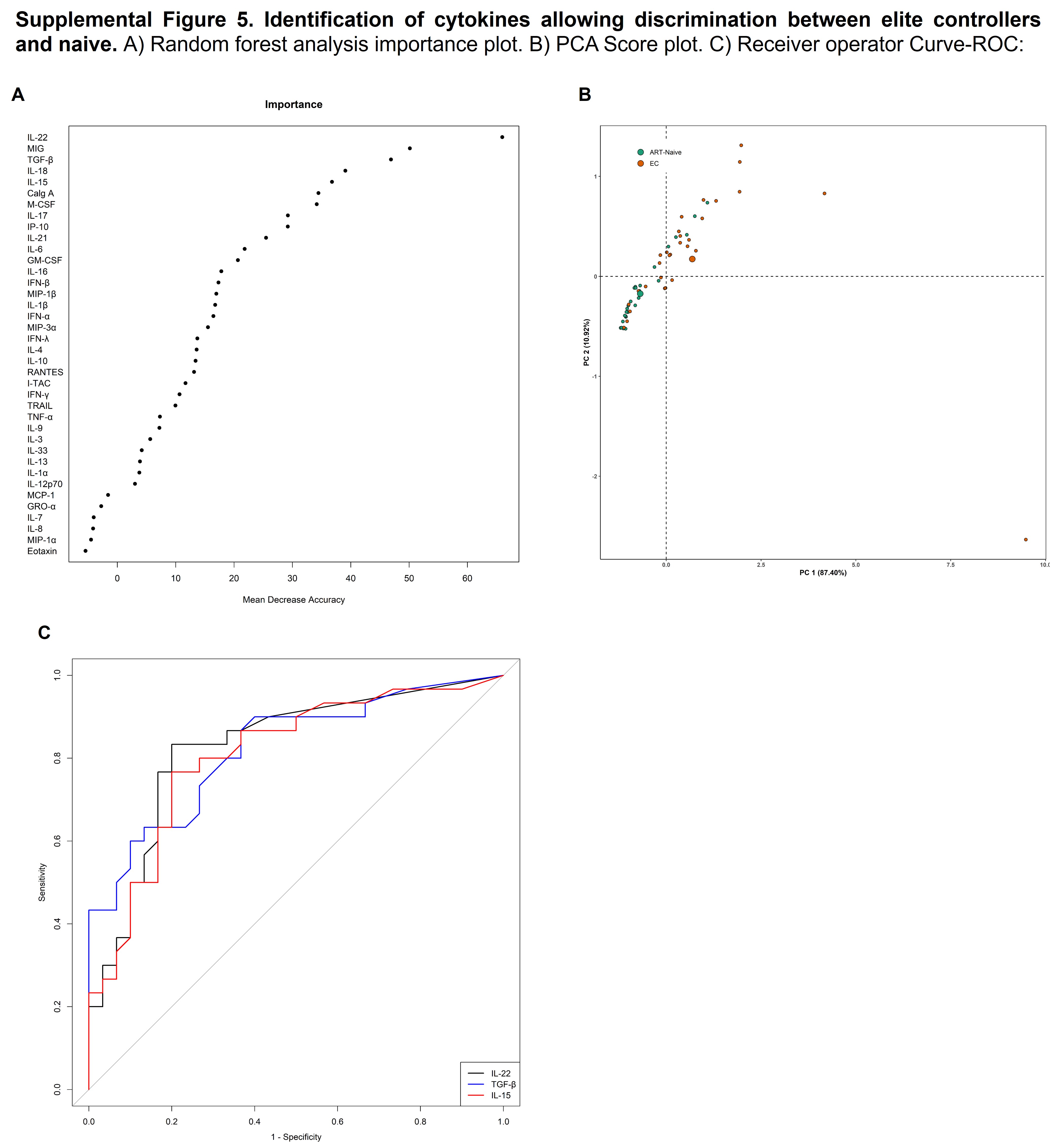

Supplement: Supplementary Figure 5 — Identification of cytokines allowing discrimination between elite controllers and naive. (A) Random forest analysis importance plot. (B) PCA Score plot. (C) Receiver operator Curve-ROC. (D) Best discriminating cytokines signatures. [file Image5.jpeg]

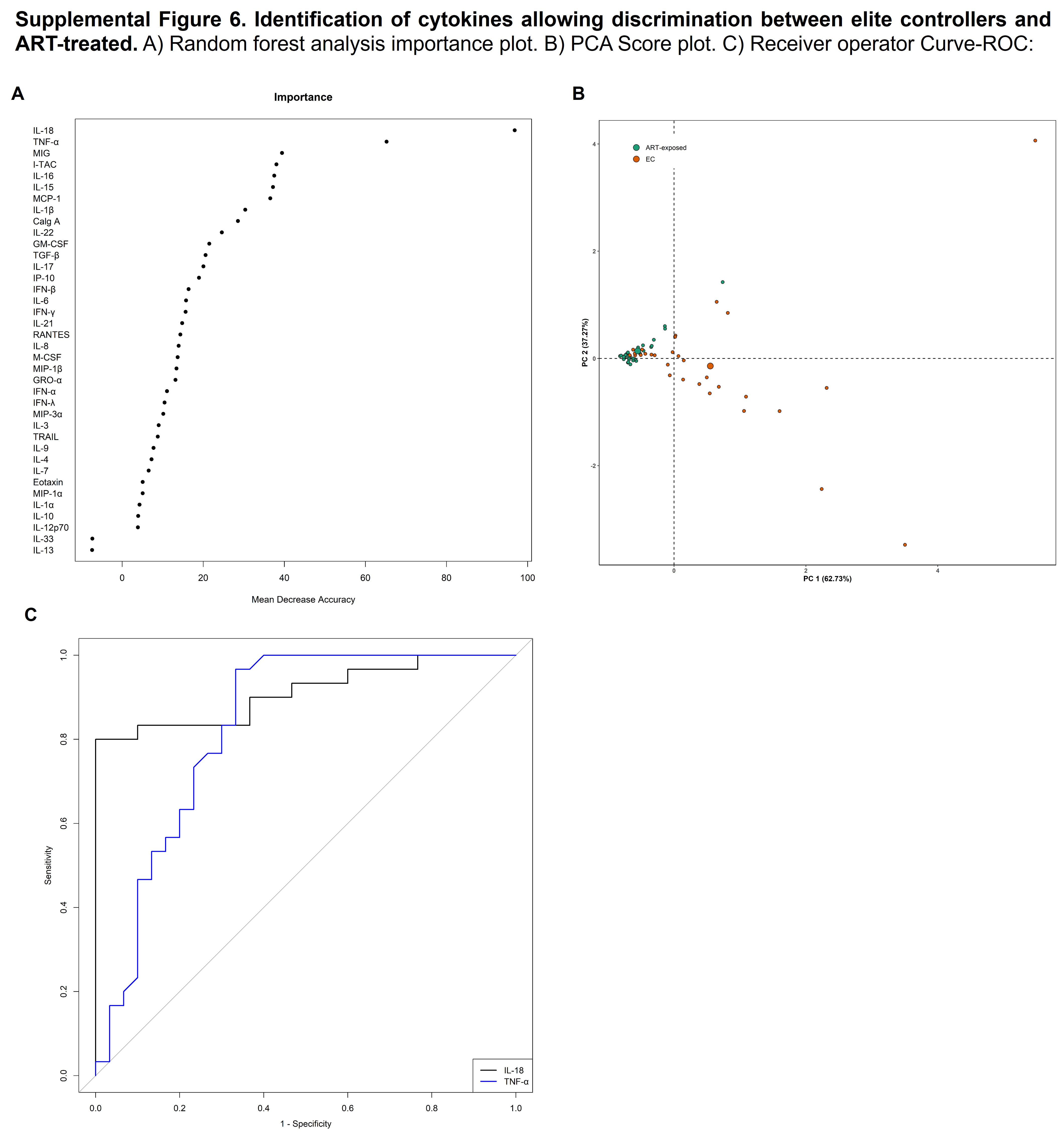

Supplement: Supplementary Figure 6 — Identification of cytokines allowing discrimination between elite controllers and ART-treated. (A) Random forest analysis importance plot. (B) PCA Score plot. (C) Receiver operator Curve-ROC. (D) Best discriminating cytokines signatures. [file Image6.jpeg]

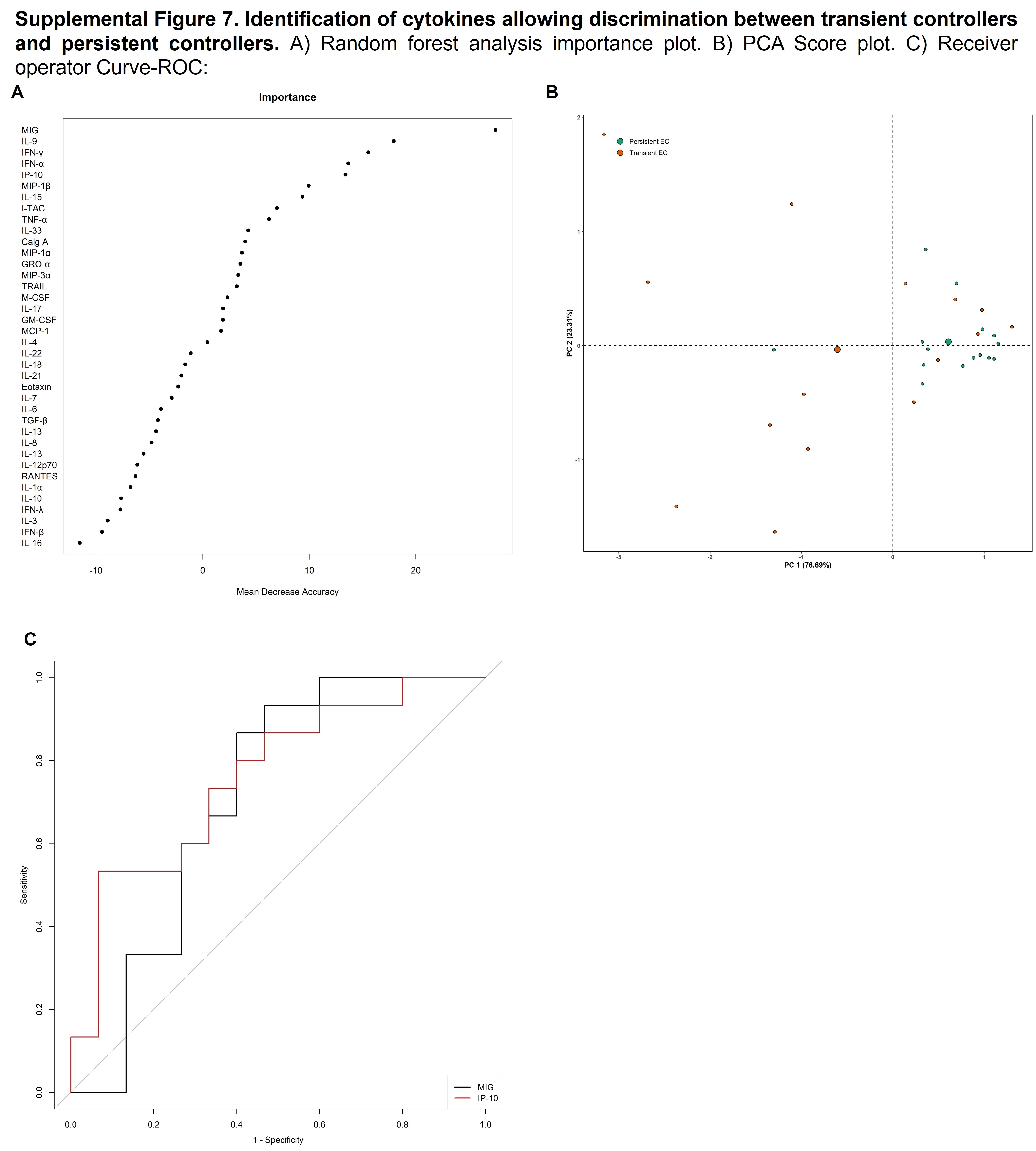

Supplement: Supplementary Figure 7 — Identification of cytokines allowing discrimination between transient and persistent controllers. (A) Random forest analysis importance plot. (B) PCA Score plot. (C) Receiver operator Curve-ROC. (D) Best discriminating cytokines signatures. [file Image7.jpeg]
