# Supplementary material for: Elevated plasma levels of IP-10 and MIG are early predictors of loss of control among elite HIV controllers
Source: Front Immunol. 2024 Aug 29;15:1446730. doi: 10.3389/fimmu.2024.1446730 (PMC11390527; doi:10.3389/fimmu.2024.1446730)
Supplement: Supplementary file 8 [file Table1.docx]

**Supplemental Table 1.** Main Characteristics of the study population.

*Abbreviations: ART, antiretroviral therapy; HIV, human immunodeficiency virus; NA, not applicable; ND, not detectable; n.s., not significant.*

*Data are expressed as median [interquartile range] and as percentage (n). Statistical significance was determined using χ^2^ test and Kruskal-Wallis test for qualitative and continuous variables, respectively.*

|  | **Control**  **(n=30)** | **ART-naïve**  **(n=30)** | **ART-treated**  **(n=30)** | **EC (PC)**  **(n=15)** | **EC (TC)**  **(n=15)** | ***p-value***  ***Global*** | ***p-value***  ***PC vs. TC*** |
| --- | --- | --- | --- | --- | --- | --- | --- |
| **Age** | 36.0  [29.0-41.0] | 41.0  [32.0-52.0] | 52.0  [43.0-56.0] | 54.1  [48-58] | 48.4  [39-52] | <0.001 | n.s |
| **Sex (male)** | 50.0 (15) | 71.0 (22) | 66.7 (20) | 53.3 (8) | 46.7 (7) | n.s. | n.s |
| **Origin** | Spain: 90 %  Rest of Europe: 10 % | Spain: 80 %  Rest of Europe: 3.3 %  South America: 13.3 % | Spain: 93.3 %  Rest of Europe: 3.3 % | Spain: 66.7 %  South America: 13.3 %  North America: 6.7 %  Africa: 6.7 % | Spain: 66.7 %  Rest of Europe: 6.7 %  South America: 20 %  North America: 6.7 % | n.a. | n.a. |
| **Year of HIV diagnosis** | NA | 2017  [2017-2018] | 2004  [1997-2011] | 1998  [1991-2006] | 1993  [1985-2003] | <0.001 | n.s |
| **Days since HIV diagnosis** | NA | 7  [0-64] | 4803  [2316-7373] | 4427  [1971-7054] | 5783  [2075-8697] | <0.001 | n.s |
| **HIV viral load (copies/ml)** | NA | 27450  [9700-110000] | ND | ND  [25-50] | ND  [37-50] | <0.001 | n.s |
| **Type of ART regimen** | NA | NA | NRTI + INSTI: 50.0 %  NRTI + NNRTI: 30.0 %  Others: 20.0 % | NA | NA | NA | NA |
| **CD4 (cells/µl)** | 842  [712-1018] | 456  [267-657] | 827  [609-1071] | 855.9  [426-1290] | 833.7  [640-1038] | <0.001 | n.s |
| **CD8 (cells/µl)** | 474  [404-653] | 983  [730-1284] | 824  [512-933] | 1075  [575-1724] | 913.9  [637-1094] | <0.001 | n.s |
| **CD4/CD8 ratio** | 1.6  [1.4-2.1] | 0.4  [0.2-0.7] | 1  [0.8-1.3] | 1.1  [0.56-1.3] | 1  [0.5-1.5] | <0.001 | n.s |
| **Platelet (cells/µl)** | 246  [211-275] | 219  [186-257] | 212  [192-245] | n.a | n.a | n.s | n.a |
| **Neutrophils (cells/µl)** | 3.40  [2.70-4.73] | 2.64  [1.91-3.21] | 3.67  [2.68-4.90] | n.a. | n.a | 0.002 | n.a |
